# Candida in the respiratory tract secretions of critically ill patients and the impact of antifungal treatment: a randomized placebocontrolled pilot trial (CANTREAT study)

**DOI:** 10.1186/cc13542

**Published:** 2014-03-17

**Authors:** M Albert, DR Willamson, J Muscedere, F Lauzier, C Rostein, S Kanji, X Jiang, M Hall, DK Heyland

**Affiliations:** 1 Hopital du Sacré-Coeur de Montréal, Canada; 2 Kingston General Hospital, Kingston, Canada; 3 Centre hospitalier affilié universitaire de Québec, Canada; 4 Toronto General Hospital, Toronto, Canada; 5 The Ottawa Hospital Research Institute, Ottawa, Canada; 6 Nationwide Children's Hospital, Colombus, OH, USA

## Introduction

Candida spp. are frequently recovered from endotracheal secretions in critically ill patients suspected of having ventilator- associated pneumonia. Observational studies reported an association with worse clinical outcomes [1] but the effect of antifungal therapy in these patients remains unclear. We designed this pilot study to assess the feasibility of a larger trial and to evaluate inflammatory profiles and clinical outcomes in these patients.

## Methods

We conducted a double-blind, placebo-controlled, multicenter, pilot randomized trial of antifungal therapy in critically ill patients with a clinical suspicion of ventilator-associated pneumonia with positive airway secretion specimens for Candida spp. We also included an observational group without Candida spp. in their airway secretions. We measured the recruitment rate, inflammatory profiles over time and clinical outcomes.

## Results

We recruited 60 patients into the randomized trial; 29 patients into the observational study. Recruitment was halted before the end of the study because of difficulty in recruiting patients. Markers of inflammation and all clinical outcomes were comparable between placebo and antifungal treatment groups at baseline and overtime. At baseline, TNFγ levels were higher in the VAP with Candida compared with the observational group (mean ± SD) (21.8 ± 23.1 vs. 12.4 ± 9.3 pg/ ml, *P *= 0.02) and these patients had lower response to the LPS stimulation test (854.8 ± 855.2 vs. 1,559.4 ± 1,290.6 pg/ml, *P *= 0.01).

## Conclusion

Given the difficulty recruiting patients and the lack of signal in clinical or inflammatory outcomes, this study does not provide evidence to support a larger trial examining the efficacy of empiric antifungal treatment in patients with a clinical suspicion of ventilator- associated pneumonia and Candida in the endotracheal secretions. The presence of Candida in the lung may be associated with persistent inflammation and immunosuppression.

## References

[B1] Delisle MS Williamson DR Perreault MM Albert M Jiang X Heyland DK The clinical significance of candida colonization of respiratory tract secretions in critically ill patients J Crit Care 2008 23 11 17 10.1016/j.jcrc.2008.01.005 18359416

